# Difference between the Upper and the Lower Gastrointestinal Bleeding in Patients Taking Nonvitamin K Oral Anticoagulants

**DOI:** 10.1155/2018/7123607

**Published:** 2018-05-15

**Authors:** Kyohei Maruyama, Takatsugu Yamamoto, Hitoshi Aoyagi, Akari Isono, Koichiro Abe, Shinya Kodashima, Hiroto Kita, Yuji Watari, Ken Kozuma

**Affiliations:** Department of Internal Medicine, Teikyo University School of Medicine, Tokyo, Japan

## Abstract

Nonvitamin K oral anticoagulants (NOACs) sometimes cause hemorrhage, and the gastrointestinal tract is a common site of involvement. However, clinical characteristics of gastrointestinal bleeding (GIB) during NOAC therapy have not been fully elucidated. We studied 658 patients who were prescribed dabigatran, rivaroxaban, or apixaban between April 2011 and November 2015. Medical charts were reviewed to examine whether clinically relevant bleeding (Bleeding Academic Research Consortium criteria type 2 or greater) developed. The incidence of GIB was 2.0%/year, and one-third was from the upper GI. Among all hemorrhagic events, GIB was the most common cause. The extent of bleeding from the GI tract, particularly the upper GI tract, was more serious than bleeding from the other site. Multiple regression analysis showed that both past digestive ulcer and absence of concomitant proton pump inhibitors were significantly associated with the incidence of upper GIB, while concomitant nonsteroidal anti-inflammatory drugs, dual antiplatelets, and past GIB were significant factors regarding lower GIB. GIB was common and serious in patients taking NOACs. Upper GIB tended to become more serious than lower GIB. Proton pump inhibitors seem to be key drugs for preventing upper GIB during NOAC therapy.

## 1. Introduction

Several nonvitamin K oral anticoagulants (NOACs) have recently been developed for preventing cardiogenic stroke in patients with atrial fibrillation. According to randomized clinical trials, NOACs show characteristics of better adherence and safety regarding adverse hemorrhagic events compared to conventional warfarin therapy [1–4]. However, concerning the risk for gastrointestinal bleeding (GIB), some researchers have indicated an increased incidence of GIB among NOAC users [5–9], while others reported the risk as closer to that of vitamin K antagonists [10–15]. Moreover, the clinical features of GIB, such as severity or bleeding site, have not yet been fully elucidated. Because the development of GIB is significantly associated with mortality in patients with atherosclerosis diseases, the precise information about GIB during NOAC therapy is important [16]. We therefore conducted this retrospective cohort study to examine the clinical manifestations of GIB in patients taking NOACs.

## 2. Materials and Methods

Study participants were selected from patients at our institution. All patients who had been prescribed dabigatran, rivaroxaban, or apixaban between April 2011 and November 2015 were identified from patient lists. Patients who had been given NOAC for nonvalvular atrial fibrillation were then selected, resulting in 658 patients (dabigatran, *n* = 220; rivaroxaban, *n* = 283; apixaban, *n* = 155) enrolled as subjects. Data were collected using the same method reported previously, as described below [17]. Medical records of subjects were examined to clarify dates of the first and last prescription and the presence or absence of hemorrhagic and thrombotic events. Endpoints were either (1) overt “actionable” bleeding (Bleeding Academic Research Consortium (BARC) types 2–5) [18]; (2) discontinuation of prescription; or (3) the end of August 2017. The clinical course was reviewed every month in each patient based on medical records, and observations ceased when the prescription was discontinued, the patient changed the medical institution that they visited periodically, or the patient stopped visiting the hospital for more than 3 consecutive months without reason (regarded as “dropout” cases). The cause of GIB was identified, where possible, from the medical records of endoscopic findings. The reason for discontinuation of NOAC was also investigated.

All statistical evaluations were made using SPSS Statistics version 19 (IBM Japan, Tokyo, Japan). Differences in the ratios or values between groups were evaluated using the chi-square test. Cox proportional hazards analysis with stepwise forward likelihood method was used in the univariate and multivariate analysis, in order to clarify significant clinical factors related to hemorrhage. A value of *p* < 0.05 was regarded as statistically significant. This protocol was approved by the institutional review board of Teikyo University prior to the study (TU-15-113-2).

## 3. Results

The background characteristics of subjects are shown in Table 1. Mean subject age at the initiation of NOAC was 72.2 years, and the ratio of males reached 68%. Dabigatran was prescribed in 33%, rivaroxaban in 43%, and apixaban in 23% of all the patients. This table also shows high rate of comorbidities and concomitant agents. Regarding antiplatelets, we confirmed that all of the concomitant prescription had been made appropriately by cardiologists or neurologists.  Table 2 shows the observational data from the study. The total observation period was 1342 patient-years. Clinically relevant bleeding was identified in 63 patients, with GIB identified in 27 patients (44%), from the upper GI in 9 patients and from the lower GI in 18 patients. The gastrointestinal tract was the most common site of bleeding. Major GIB (BARC type 3 or above) was found in 12 of the 27 patients with GIB (43%), slightly higher than bleeding from any other site (10/36, 28%), although the difference remained nonsignificant (*p* = 0.170, chi-squared test). In particular, severity of bleeding was significantly higher in the upper GIB than in the lower GIB (*p* = 0.014, chi-squared test) (Table 2).

The common cause of major upper GIB was digestive ulcer, and no patients were taking PPI concomitantly (*p* = 0.003, chi-squared test) (Table 3). Most of the major upper GIB occurred 1 year or more after initiating NOACs.

Table 4 showed the significance of clinical factors with the upper and the lower GIB evaluated be univariate analysis. Several factors were significantly associated with GIB. A novel HAS-BLED score for risk assessment of anticoagulant therapy showed significant association with the lower GIB, but not with the upper GIB. Multiple regression analysis using all of the factors in Table 4 showed that factors significantly related to upper GIB included use of PPI and past history of digestive ulcers (Table 5).

Common causes of lower GIB were telangiectasia and hemorrhoids, most of which were not clinically serious. These cases of GIB occurred relatively early after starting NOAC therapy. Clinical factors relating to lower GIB were concomitant use of NSAIDs and dual antiplatelets, past GIB, and female sex (Table 6).

## 4. Discussion

The present study shows the current status of gastrointestinal hemorrhagic events in patients taking NOACs at a single Japanese institution. Because little detailed information about GIB during NOAC therapy has been reported for Asian populations, the present data should be helpful for clinicians who encounter patients originally from Asian countries.

The incidence of all bleeding events in the present study was calculated as 4.7%/year, and that of clinically significant GIB was 2.0%/year, with upper GIB comprising 0.9%/year and lower GIB comprising 1.1%/year. The rate of lower GIB was comparable to those reported in previous studies, whereas that of upper GIB seemed lower. Miller et al. reported upper GIB in 1.5% and lower GIB in 1.0% in a review of 43 clinical trials comprising over 160,000 patients [10]. This difference in the rate of upper GIB may be explained by the high rate of concomitant PPI. The rate of PPI prescription reached 47% in this study, while Chan et al. reported PPI as one factor significantly related to upper GIB during NOAC therapy, where the prescription rate remained below 20% [19]. Another reason may be the older mean age of the present subjects. Mean age was around 72 years old, and most lower GIB developed in elderly patients of 75 years old or more, whereas patients in most previous studies were younger than 70 years old.

The present data indicate that the characteristics of GIB might differ between the upper and lower gastrointestinal tracts. Concerning the upper digestive tract, GIB often developed to a serious condition requiring hospitalization and transfusion (Table 3). Notably, most cases of major upper GIB developed from peptic ulcer diseases in patients not taking PPI concomitantly. Because most such patients had a past history of peptic ulcer, elicitation of any history of peptic ulcer is extremely important when considering initiation of NOACs. Esophagogastroduodenoscopy is highly recommended in such cases.

The incidence of lower GIB was higher than that of upper GIB. Most of lower GIB, however, was nonmajor bleeding that did not need specific medical intervention. In addition, these events typically occurred in the early period after initiating NOAC. Significant factors of the lower GIB included concomitant NSAIDs and dual antiplatelets. Concomitant use of anticoagulants and NSAIDs or antiplatelets has been reported to increase the risk of GIB. Kumar et al. showed that triple therapy with NOAC, aspirin, and clopidogrel resulted in a 2.5-fold increase in GIB [20]. Concerning NSAIDs, Lamberts et al. showed NSAIDs to be an independent risk factor for serious hemorrhage in patients with antithrombotic agents [21]. In any case, clinicians should pay attention to concomitant use of NOACs, and avoiding such use is desirable. Other significant factors relating to lower GIB were female sex and history of GIB. Why female sex had an impact on GIB was unclear, but the difference in the average age of subjects between sexes might be involved.

Differences among different NOACs have been reported. Results accumulated from RCTs have implied that dabigatran and rivaroxaban might carry a higher risk of GIB than other NOACs [6]. A recent meta-analysis regarding data in clinical settings also supported this tendency [10]. In the present study, however, little difference in the incidence of GIB was seen among NOACs. One possible hypothesis regarding the difference between previous findings and our data suggests that clinicians might have paid close attention to such vulnerable patients when prescribing rivaroxaban in clinical settings, which might in turn have lowered the incidence of GIB. This possibility might be supported by the fact that the present patients taking dabigatran or rivaroxaban were significantly younger than those taking apixaban (dabigatran; 70.2 ± 9.8 years, rivaroxaban; 72.1 ± 9.5, apixaban; 75.6 ± 10.4, *p* < 0.01, by unpaired *t*-test). Nevertheless, although further follow-up studies are needed to elucidate the actual significance of these differences, the present data showed that the difference might be small.

Some limitations must be considered when interpreting the present data. One was the retrospective study design. Further prospective cohort studies are required to clarify the real risks for NOAC-related GIB. Second, a total of 12 patients dropped out. They might have disappeared because of serious events such as bleeding. Finally, this study was conducted at a single institution. A multi-institution study is needed to confirm the current situation in Japan.

## Figures and Tables

**Table 1:** Background characteristics of the patients.

Clinical factor	Number of patients
Biographic data	
Age	72.2 ± 10.0 years
Sex (male)	448 (68.1%)
Weight	63.0 ± 13.9 kg
Serum creatinine	0.95 ± 0.31 mg/dl
CHADS2 score	2.5 ± 1.2
HAS-BLED score	2.1 ± 0.9
NOAC	
Dabigatran	220 (33.4%)
Rivaroxaban	283 (43.0%)
Apixaban	155 (22.8%)
Past history	
GIB	7 (1.1%)
Digestive ulcer	25 (3.8%)
Cerebral infarction	92 (14.0%)
Coexistent disease	
Hypertension	553 (84.0%)
Diabetes mellitus	167 (25.4%)
Dyslipidemia	295 (44.8%)
Chronic heart failure	403 (61.2%)
Malignant diseases	64 (9.7%)
Concomitant agent	
Low dose aspirin	127 (19.3%)
Thienopyridine	68 (10.3%)
Dual antiplatelet	28 (4.3%)
NSAIDs	13 (2.0%)
Steroids	30 (4.6%)
Diuretics	165 (25.1%)
BP	16 (2.4%)
PPI	313 (47.6%)
H2RA	39 (5.9%)
MP	46 (7.0%)

NOAC: nonvitamin K oral anticoagulant, GIB: gastrointestinal bleeding, NSAID: nonsteroidal anti-inflammatory drugs, BP: bisphosphonate, PPI: proton pump inhibitor, H2RA: histamine 2 receptor antagonist, and MP: mucoprotective agent.

**Table 2:** Observational data.

	Number of patients
	(%/year)
Number of patients	658
Observational period	1342.2 patient-year
Event	
Total bleeding (≧BARC type 2)	63 (4.7)
Major bleeding (≧BARC type 3)	22 (1.6)
Total GIB	27 (2.0)
Upper GIB	9 (0.7)
Lower GIB	18 (1.3)
Major GIB (≧BARC type 3)	12 (0.9)
Fatal bleeding	3 (0.2)
Thrombosis	16 (1.2)
Dropout	12 (0.9)

GIB: gastrointestinal bleeding and BARC: bleeding academic research consortium classification.

**Table 3:** Cases of major gastrointestinal bleeding in patients taking direct oral anticoagulants.

Age, sex	NOAC	Lesion	Prescription (month)	BARC	Other factors
Upper GI					
92 F	D	Gastric ulcer	11	3a	PPI (-)
96 F	R	Gastric ulcer	42	3a	PPI (-)
63 M	R	Duodenal ulcer	29	3b	PPI (-)
74 M	R	Gastric ulcer	32	3a	PPI (-)
83 F	A	Gastric ulcer	16	3a	PPI (-)
84 M	A	Ulcer s/o	12	3a	PPI (-), steroid
72 M	A	Duodenal ulcer	25	3b	PPI (-), operation
Lower GI					
78 M	D	Ileal erosion	23	3a	DAPT
80 M	D	Colon diverticulosis	7	3a	-
72 F	R	Colon vascular ectasia	8	3a	NSAID, steroid
75 M	R	Post EMR for colon polyp	24	3a	LDA
85 F	A	Colon diverticulosis	3	3b	NSAID

Major bleeding means bleeding greater than Bleeding Academic Research Consortium classification type 3. NOAC: nonvitamin K oral administrative drugs, F: female, M: male, GI, gastrointestinal, D: dabigatran, R: rivaroxaban, A: apixaban, PPI: proton pump inhibitor, DAPT: double antiplatelet therapy, EMR: endoscopic mucosal resection, and NSAID: nonsteroidal anti-inflammatory drug.

**Table 4:** Significance of clinical factors and gastrointestinal bleeding in patients taking nonvitamin K oral anticoagulants (univariate analysis).

Clinical factor	Upper GIB	*p* value^*∗*^	Lower GIB	*p* value^*∗*^
	HR (95% CI)		HR (95% CI)	
Biographic data				
Age	**1.13 (1.03**–**1.23)**	**0.01**	**1.06 (1.00**–**1.13)**	**0.03**
Sex	0.89 (0.22–3.54)	0.86	**0.36 (0.14**–**0.90)**	**0.03**
Weight	0.97 (0.92–1.03)	0.30	**0.95 (0.91**–**0.99)**	**0.02**
Serum creatinine	1.43 (0.26–7.89)	0.69	0.79 (0.13–4.62)	0.79
CHADS2 score	0.94 (0.54–1.64)	0.82	1.06 (0.72–1.57)	0.76
HAS-BLED score	0.84 (0.40–1.77)	0.64	**2.21 (1.35**–**3.61)**	**<0.01**
NOAC				
Dabigatran	0.24 (0.03–1.91)	0.18	1.26 (0.46–3.45)	0.65
Rivaroxaban	1.09 (0.29–4.06)	0.90	1.07 (0.40–2.86)	0.89
Apixaban	2.73 (0.71–10.5)	0.14	0.66 (0.19–2.33)	0.52
Past history				
GIB	0.05 (0–4E + 12)	0.85	**15.9 ** **(** **3.6**–**70.0)**	**<0.01**
Digestive ulcer	**16.8 (4.5**–**62.6)**	**<0.01**	0.05 (0–1E + 4)	0.56
Cerebral infarction	0.71 (0.09–5.65)	0.74	0.36 (0.05–2.70)	0.32
Coexistent disease				
Hypertension	24.6 (0–1E + 6)	0.48	1.15 (0.26–5.03)	0.86
Diabetes mellitus	0.39 (0.05–3.10)	0.85	0.85 (0.28–2.60)	0.78
Dyslipidemia	0.14 (0.02–1.14)	0.07	2.24 (0.84–5.98)	0.11
Chronic heart failure	1.15 (0.29–4.61)	0.85	1.17 (0.43–3.12)	0.76
Malignant diseases	0.04 (0–724)	0.53	0.04 (0–37.6)	0.36
Concomitant agent				
Low dose aspirin	0.03 (0–32.4)	0.34	**3.03 (1.19**–**7.68)**	**0.02**
Thienopyridine	0.04 (0–1E + 4)	0.56	2.93 (0.95–9.04)	0.06
Dual antiplatelet	0.05 (0–1E + 6)	0.69	**7.19 (2.34**–**22.1)**	**<** **0** **.01**
NSAIDs	0.05 (0–4E + 9)	0.81	**10.19 (2.70**–**38.4)**	**<0.01**
Steroids	2.17 (0.27–17.4)	0.47	1.22 (0.16–9.21)	0.85
Diuretics	1.43 (0.36–5.71)	0.61	1.39 (0.52–3.71)	0.51
BP	4.59 (0.57–36.8)	0.15	**5.16 (1.18**–**22.6)**	**0.03**
PPI	0.12 (0–3.46)	0.13	1.96 (0.74–5.24)	0.18
H2RA	1.60 (0.2–12.9)	0.66	1.83 (0.41–8.0)	0.43
MP	0.04 (0–1E + 4)	0.57	2.20 (0.63–7.69)	0.22

^*∗*^Analyses were done by Cox proportional hazard model with stepwise forward likelihood method. NOAC: nonvitamin K oral anticoagulant, GIB: gastrointestinal bleeding, NSAIDs: nonsteroidal anti-inflammatory drugs, BP: bisphosphonate, PPI: proton pump inhibitor, H2RA: histamine 2 receptor antagonist, and MP: mucoprotective agent.

**Table 5:** Significant clinical factors relating upper gastrointestinal bleeding in patients taking nonvitamin K oral anticoagulants.

Factor	Adjusted HR (95% CI)	*p* value
PPI	0 (0–2E + 134)^*∗*^	<0.001
Past digestive ulcer	29.114 (7.265–116.678)	<0.001

Analysis was done by Cox proportional hazard model with stepwise forward likelihood method. ^*∗*^Adjusted HR of PPI presents 0 because no patients with upper gastrointestinal bleeding took PPI concomitantly. HR: hazard ration, CI: confidence interval, and PPI: proton pump inhibitor.

**Table 6:** Significant clinical factors relating lower gastrointestinal bleeding in patients taking nonvitamin K oral anticoagulants.

Factor	Adjusted HR (95% CI)	*p* value
NSAIDs	12.6 (3.2–49.1)	<0.001
Dual antiplatelet	8.6 (2.7–27.1)	<0.001
Past GIB	15.1 (3.2–72.0)	0.001
Female	3.2 (0.1–0.8)	0.019

Analysis was done by Cox proportional hazard model with stepwise forward likelihood method. HR: hazard ratio, CI: confidence interval, NSAIDs: nonsteroidal anti-inflammatory drugs, and GIB: gastrointestinal bleeding.
